# Cancer-of-Unknown-Primary-Origin: A SEER–Medicare Study of Patterns of Care and Outcomes among Elderly Patients in Clinical Practice

**DOI:** 10.3390/cancers14122905

**Published:** 2022-06-13

**Authors:** Linda Mileshkin, Tilmann Bochtler, Gemma Gatta, Razelle Kurzrock, Andreas Beringer, Mathis Müller-Ohldach, Andy Surinach, Camille Perret, Marlene Thomas, Adam Gondos, Alwin Krämer

**Affiliations:** 1 Department of Medical Oncology, Peter MacCallum Cancer Centre and Sir Peter MacCallum Department of Oncology, The University of Melbourne, Melbourne, VIC 3000, Australia; 2 Clinical Cooperation Unit Molecular Hematology/Oncology, German Cancer Research Center (DKFZ) and Department of Internal Medicine V, University of Heidelberg, Im Neuenheimer Feld 280, 69120 Heidelberg, Germany; tilmann.bochtler@med.uni-heidelberg.de (T.B.); a.kraemer@dkfz-heidelberg.de (A.K.); 3 Department of Medical Oncology, National Center for Tumor Diseases (NCT), Heidelberg University Hospital, Im Neuenheimer Feld 460, 69120 Heidelberg, Germany; 4 Evaluative Epidemiology Unit, IRCCS Foundation, National Cancer Institute, Via Venezian 1, 20133 Milan, Italy; gemma.gatta@istitutotumori.mi.it; 5 Center for Personalized Cancer Therapy and Division of Hematology and Oncology, UC San Diego Moores Cancer Center, 3855 Health Sciences Drive, La Jolla, CA 92037, USA; rkurzrock@mcw.edu; 6 Global Product Development Medical Affairs, F. Hoffmann-La Roche Ltd., Building 1, Grenzacherstrasse 124, CH-4070 Basel, Switzerland; andreas.beringer@roche.com; 7 PDMA Medical Alliances Operations, F. Hoffmann-La Roche Ltd., Building 663, U1.312 Hochstrasse 16, CH-4053 Basel, Switzerland; mathis.mueller-ohldach@roche.com; 8 Evidence Strategy, Genesis Research, 111 River Street, Suite 1120, Hoboken, NJ 07030, USA; andy@genesisrg.com; 9 Oncology, Personalised Healthcare, F. Hoffmann-La Roche Ltd., Building 1, Grenzacherstrasse 124, CH-4070 Basel, Switzerland; camille.perret@roche.com; 10 Global Medical Affairs/PDMA, F. Hoffmann-La Roche Ltd., Building 1, Grenzacherstrasse 124, CH-4070 Basel, Switzerland; marlene.thomas@roche.com; 11 Pharmaceutical Division, F. Hoffmann-La Roche Ltd., Building 1, Grenzacherstrasse 124, CH-4070 Basel, Switzerland; adam.gondos@roche.com

**Keywords:** diagnostic tests and procedures, drug therapy, Medicare Part A, Medicare Part B, neoplasms, registries, SEER program, survival analysis, unknown primary

## Abstract

**Simple Summary:**

Cancer-of-unknown-primary-origin (CUP) is challenging to diagnose and treat, and little is known about its diagnostic work-up, treatment, and outcomes in routine healthcare. We examined data from elderly patients (at least 66 years old) diagnosed with CUP in real-world US clinical practice, using the Surveillance, Epidemiology, and End Results–Medicare-linked database. Only half of elderly patients with CUP received all three diagnostic work-up procedures (biopsy, immunohistochemistry, and imaging), as recommended by guidelines. Patients who received all three diagnostic work-up procedures were more likely to receive any type of anticancer treatment, and patients who did not receive full diagnostic work-up had shorter median overall survival, particularly with increasing age. Overall, these results suggest that further studies are needed to understand why many patients given a diagnosis of CUP do not receive complete diagnostic work-up or treatment. Further research into improving diagnostic work-up and treatment effectiveness in patients diagnosed with CUP is required.

**Abstract:**

Knowledge of contemporary patterns of cancer-of-unknown-primary-origin (CUP) diagnostic work-up, treatment, and outcomes in routine healthcare is limited. Thus, we examined data from elderly patients diagnosed with CUP in real-world US clinical practice. From the Surveillance, Epidemiology, and End Results–Medicare-linked database, we included patients ≥ 66 years old with CUP diagnosed between 1 January 2013 and 31 December 2015. We analyzed baseline demographics, clinical characteristics, methods of diagnostic work-up (biopsy, immunohistochemistry, imaging), treatment-related factors, and survival. CUP diagnosis was histologically confirmed in 2813/4562 patients (61.7%). Overall, 621/4562 (13.6%) patients received anticancer pharmacotherapy; among these, 97.3% had a histologically confirmed tumor and 83.1% received all three procedures. Among those with a histologically confirmed tumor, increasing age, increasing comorbidity score, not receiving all three diagnostic measures, and having a not-further specified histologic finding of only ‘malignant neoplasm’ were all negatively associated with receipt of anticancer pharmacotherapy. Median overall survival was 1.2 months for all patients. Median time between CUP diagnosis and treatment initiation was 41 days. Limited diagnostic work-up was common and most patients did not receive anticancer pharmacotherapy. The poor outcomes highlight a substantial unmet need for further research into improving diagnostic work-up and treatment effectiveness in CUP.

## 1. Introduction

Cancer-of-unknown-primary-origin (CUP) describes a heterogeneous group of metastatic cancers, without an identifiable primary tumor despite thorough clinical work-up [1]. Although some reports highlight a decline in incidence [2,3], likely due to improvements in primary tumor diagnostics [2,3,4], CUP still accounts for 2–5% of all malignancies [2,3,5]. Recognized based on clinical and pathologic criteria [6], around 15–20% of patients belong to favorable subsets, which may allow for site-specific treatment [6], while 80–85% of patients have a strikingly poor prognosis and a median survival of approximately 1 year or less [3,5,6], making CUP the third to fourth most common cause of cancer-related death [7]. Poor clinical outcomes likely reflect the per se metastatic state at diagnosis and inherently aggressive biology of CUP [4]. In addition, patients are often elderly, have limited standard treatment options available, and respond poorly to empiric chemotherapy [8].

International clinical practice guidelines (e.g., European Society for Medical Oncology [ESMO] [6], National Institute for Health and Care Excellence [NICE] [9], Spanish Society for Medical Oncology [SEOM] [10]) recommend thorough diagnostic work-up consisting of medical history, complete physical examination, biopsy with immunohistochemistry analysis, laboratory tests, as well as computed tomography or magnetic resonance imaging of the thorax, abdomen, and pelvis. Genomic analysis, including next-generation sequencing (NGS), is also increasingly employed to delineate a putative primary tumor site or to select patients for potential targeted therapy regardless of primary site [11,12,13,14,15]. Accordingly, National Comprehensive Cancer Network (NCCN) guidelines now recommend considering NGS to identify potentially actionable genomic alterations in patients with CUP [16].

Few studies are available that provide a detailed analysis of diagnostic work-up of CUP in population-based cancer registries (i.e., registry-notified CUP) [17] and, consequently, very little is known about contemporary real-world patterns of diagnostic work-up, treatment, and outcomes outside clinical trials. Among elderly patients (≥66 years of age) with registry-notified CUP from the US Surveillance, Epidemiology, and End Results (SEER)–Medicare (SEERM)-linked database, the specific aims of this study were to: (1) describe the demographic and clinical characteristics; (2) examine the use of diagnostic work-up; and (3) assess treatments and outcomes.

## 2. Materials and Methods

### 2.1. Study Population

This study comprised population-based US cancer registry data from the SEER Program with linked Medicare claims of billed healthcare services. Patients were included if a CUP diagnosis (including all neoplasms of unknown primary) was registered as their first malignancy at age ≥ 66 years between 1 January 2013 and 31 December 2015, according to coding criteria in the SEERM database (cancer registry coded International Classification of Diseases for Oncology, 3rd Edition [ICD-O-3] topography C80.9 [18], excluding morphology codes 9050–9055, 9140, and 9590–9992). Patients were excluded: if CUP was identified exclusively by death certificate or autopsy; if they were diagnosed with a subsequent (separate primary) cancer following their diagnosis of CUP; if they had <12 months Medicare health insurance Part A & B enrollment (federal health insurance system providing coverage for the elderly in the US, including inpatient, doctor’s services, and outpatient cover) prior to CUP diagnosis, or up until 31 December 2016 post-CUP diagnosis, or until death if that occurred earlier; or if they had a Medicare cancer treatment claim prior to their CUP diagnosis (Tables S1 and S2).

### 2.2. Analyses

#### 2.2.1. Baseline Patient Demographics

Demographic and clinical characteristics analyzed at CUP diagnosis included age, sex, ethnicity, date of diagnosis, and National Cancer Institute (NCI) comorbidity score [19]. The date of diagnosis was based on registry data, with the 15th of the month imputed for all patients (an exact day was not provided by the registry for confidentiality reasons). Histologic confirmation was defined, according to SEER diagnostic confirmation classification [20], as positive histology (for a malignant tumor) only, positive cytology only, or positive histology plus positive immunophenotyping and/or positive genetic studies. Non-histologic confirmation was defined as identification via imaging only (radiology/other imaging techniques without histologic confirmation), other (positive laboratory test/marker study; direct visualization without histologic confirmation), or clinical diagnosis only (other than imaging only or other).

The NCI comorbidity score is used to predict non-cancer-related deaths in patients with cancer via SEERM data. It includes disease conditions determined from inpatient and physician claims for a 365-day period prior to the month of diagnosis [19].

#### 2.2.2. Diagnostic Work-Up

We assessed the use of diagnostic work-up between 52 weeks prior to CUP diagnosis and up to 30 days thereafter, or up to the start of treatment if that occurred earlier. Based on ESMO and NCCN guidelines [6], a baseline diagnostic work-up of CUP was considered to require at least: (i) a biopsy; (ii) immunohistochemistry; and (iii) an imaging modality of the thorax, abdomen, and pelvis, specific for cancer diagnostics (computed tomography, positron emission tomography, or magnetic resonance imaging). Our outcome measure for diagnostic work-up was based on whether a diagnostic procedure had been performed at least once, and patients were categorized by whether they had received: (a) none of the three examinations; (b) only imaging; (c) only biopsy; (d) only biopsy and immunohistochemistry; (e) only imaging and biopsy; or (f) all three examinations.

#### 2.2.3. Treatment

For treatment-related factors, use of anticancer pharmacotherapy, as well as cancer surgery and radiotherapy, time to treatment initiation, and number of days where treatment was received (unique days with treatment administered in the follow-up period, not duration of treatment; only days where patients had received anticancer pharmacotherapy treatment were included) were described over the complete follow-up period. Median and percentage survival at 1, 2, 6, 12, 24, 36, and 60 months after diagnosis, using Kaplan–Meier estimates stratified by age groups (66–74, 75–84, and ≥85) and according to receipt of anticancer pharmacotherapy, were calculated with comparisons conducted using log-rank tests to produce *P* values. To further examine the role of diagnostic work-up in the receipt of pharmacotherapy, multivariate logistic regression was used to adjust for differences in baseline demographics (age, sex, race, year of diagnosis, as well as area-level poverty, and urbanicity indicators) and clinical characteristics (NCI comorbidity score, histology) between patients who received anticancer pharmacotherapy and those who did not. Further information regarding the compilation of study-related code lists is provided in the Supplementary Material.

## 3. Results

Overall, 6116 patients aged ≥66 years old had a first diagnosis of CUP between 1 January 2013 and 31 December 2015, of which 4562 (74.6%) patients were eligible (Table S1). Among those excluded, 657 (10.7%) had CUP identified by death certificate or autopsy only, and 578 (9.5%) had <12 months of Medicare Part A & B enrollment prior to CUP diagnosis.

### 3.1. Patient Demographics and Clinical Characteristics

Median age at first CUP diagnosis was 80 years (interquartile range: 73–86 years; Table 1). There were slightly more female than male patients (2453/4562 [53.8%] versus 2109/4562 [46.2%]). Most patients were White (3936/4562; 86.3%) and fully urban-based (2663/4562 [58.4%]). By end of study follow-up on 31 December 2016, 4163/4562 patients (91.3%) had died.

Within the study cohort, 1446/4562 patients (31.7%) had an NCI comorbidity score of zero, whereas low-, middle-, and high-level scores were each recorded for ~20–25% of patients (Table 1). A comorbidity score of zero was more frequent among patients treated with anticancer pharmacotherapy compared with those that were not (302/621 [48.6%] versus 1144/3941 [29.0%], respectively). CUP diagnosis was histologically confirmed in 2813/4562 patients (61.7%) overall (Table 1), with little variation in respective relative proportions by race, urbanicity, or census tract poverty indicator (Table 1). Pathologic confirmation was based on positive histology in 2316/2813 (82.3%) patients, and positive cytology only in 495/2813 (17.6%) patients (method not specified: 2/2813 [<0.1%]). Most common histologic types identified included adenocarcinoma (1149/2813 [40.8%]) and carcinoma not otherwise specified (479/2813 [17.0%]).

### 3.2. Characteristics of Diagnostic Work-Up

**Figure 1:**
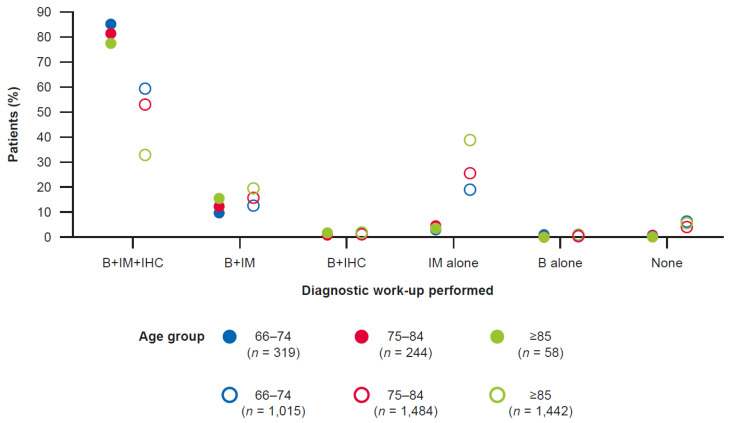
Trends in the number of diagnostics^a^ performed among treated (*n* = 621; closed circles) and untreated patients (*n* = 3941; open circles) with CUP. B, biopsy; CUP, cancer-of-unknown-primary-origin; IHC, immunohistochemistry; IM, imaging. ^a^ Record of claim for a biopsy, immunohistochemistry, or imaging modality (computed tomography, positron emission tomography, magnetic resonance imaging) for any reason.

As indicated by the volume of claims for biopsies, immunohistochemistry, and imaging (Figure S1), diagnostic work-ups were mostly completed in the few weeks prior to, and at the time of, CUP diagnosis and continued, albeit in a decreasing fashion, in the first few weeks following CUP diagnosis. Among patients who received anticancer pharmacotherapy, 516/621 (83.1%) received all three diagnostic work-up procedures at least once, with little variation by age (Figure 1 and Table S3). Among untreated patients, only 1865/3941 (47.3%) received all three procedures, more commonly in younger patients; 1133/3941 (28.7%) patients received imaging only (Figure 1 and Table S3).

### 3.3. Treatment

Of the 4562 patients in the study, only 621 (13.6%) received any anticancer pharmacotherapy (Table 2). Among those treated, 604 (97.3%) had a histologically confirmed tumor (Table 1). Of those treated with anticancer pharmacotherapy, having more than one line of treatment was uncommon; among the 621 patients who received treatment, 283 (45.6%) received either just one single day of treatment (18.4%) or only 2–4 days (27.2%). The use of both radiotherapy and cancer surgery were more frequent compared with those who did not receive pharmacotherapy (255/621 [41.1%] versus 300/3941 [7.6%], and 100/621 [16.1%] versus 190/3941 [4.8%]), respectively; Table 2). The median number of days between CUP diagnosis and start of treatment was 41 days (range: 37–44 days; Table 2).

### 3.4. Receipt of Anticancer Pharmacotherapy

Over the entire course of treatment, the majority of patients treated with pharmacotherapy received chemotherapy only (369/621 [59.4%]), while 22 (3.5%) received cancer immunotherapy (Table 2). Targeted therapies were recorded for 99/621 (15.9%) of the treated patients, with bevacizumab and cetuximab the most commonly prescribed agents (data not shown). According to the multivariate analysis, not receiving all three main diagnostic measures at least once was found to be negatively associated with the receipt of anticancer pharmacotherapy (Table 3), as were increasing age, increasing comorbidity score, increasing poverty, and having a histologic finding of malignant neoplasm as opposed to a more specific diagnosis such as adenocarcinoma (Table 3). In contrast, having a histologic diagnosis of squamous, neuroendocrine, or small-cell carcinomas was associated with an increased likelihood of receiving anticancer pharmacotherapy, compared with adenocarcinoma.

### 3.5. Survival Analysis

**Figure 2:**
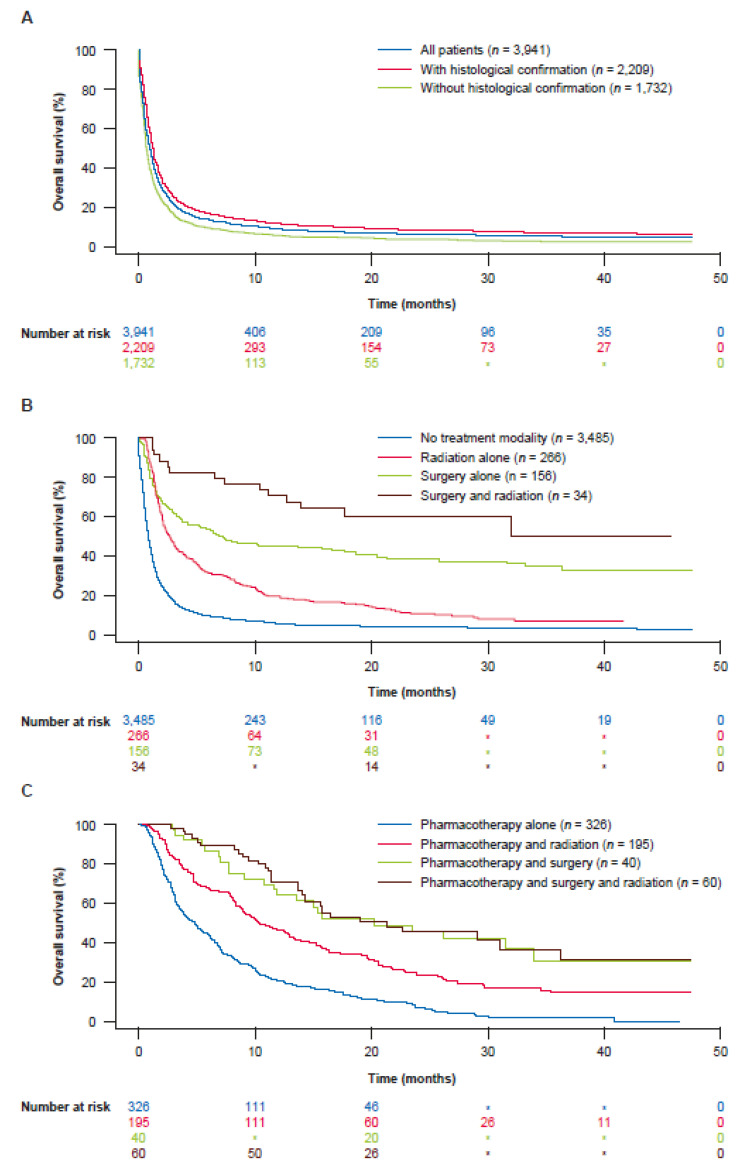
Survival of patients with CUP who were (**A**) untreated, overall and by histologic confirmation status, (**B**) treated without pharmacotherapy, and (**C**) treated alongside pharmacotherapy. * Suppressed data based on NCI guidelines for confidentiality. CUP, cancer-of-unknown-primary-origin; NCI, National Cancer Institute. See Table S4 for further data on patient survival.

Survival estimates for patients from CUP diagnosis date, overall and by treatment, age, selected clinical factors, and number of treatment days are shown in Tables S4 and S5. Median overall survival was 1.2 months and the percentage of patients still alive after 6 months, 1 year, and 3 years was only 20.3%, 13.7%, and 7.1%, respectively (Figure 2 and Table S4). The median survival was 9.5 months (95% CI: 8.2–10.5) and 1.0 month (95% CI: 0.9–1.0; *p* < 0.0001) for treated and untreated patients, respectively. The highest 3-year survival (50.5% [*n* = 34]) was observed for patients who were treated with surgery and radiotherapy only (Figure 2). Lowest 3-year survival (2.5% [*n* = 1679]) was seen among patients who did not receive histologic confirmation of their tumor and were not treated (Figure 2). Among those who received pharmacotherapy, median overall survival was longer in those who received more days of treatment (Table S5).

## 4. Discussion

To our knowledge and based on the inclusion of diagnostic work-up treatment-related information from medical service claims and its use to examine the three key aspects of diagnostic work-up, this is the most comprehensive SEERM study to date among elderly patients (≥66 years old) diagnosed with CUP in real-world clinical practice.

Only half of elderly patients with CUP received all three diagnostic work-up procedures (biopsy, immunohistochemistry, and imaging) as recommended by the ESMO/NCCN guidelines [6,16]. Our findings are in keeping with results seen in other CUP-SEERM studies, where 22–65% of patients had limited diagnostic work-up and often lacked a histologic evaluation [21,22,23]. Notably, patients without a histologic diagnosis including immunohistochemistry or imaging do not meet the requirements for diagnosis of CUP by ESMO/NCCN criteria [6,16]. It is unclear whether patients with limited diagnostic work-up had a ‘true’ CUP diagnosis, or a more treatable malignancy or some other pathology, especially when based on imaging alone. Accordingly, the SEERM database and current study cohort likely include both patients with a ‘true’ CUP diagnosis and patients with a ‘provisional’ default diagnosis of CUP without a full diagnostic work-up.

While studying CUP using cancer registry-based [24,25] as well as linked cancer registry and claims data [17,26,27] provides important insights into the clinical features and care of these patients, challenges remain due to the lack of thorough clinical details and ability to delineate a clinically validated CUP population in cancer registries. CUP-specific, clinically tailored, and detailed databases would help promote further understanding of the disease. Existing challenges regarding CUP in routine clinical practice stress the need to define CUP diagnosis as fully as possible, and to verify each CUP diagnosis meticulously in the eligibility process of clinical trials to obtain a ‘true’ CUP cohort and exclude more easily treatable cancers, as exemplified by screening challenges in the CUPISCO trial (NCT03498521) [28].

In our study, patients with complete baseline diagnostic work-up were more likely to receive any type of anticancer treatment, suggesting that a complete diagnostic work-up was mostly regarded as mandatory before initiating therapy. In particular, the best survival outcomes were seen in those patients determined to belong to a subset with good prognosis, such as those with a single site of disease who may be treated with surgery and/or radiotherapy. Conversely, omitting diagnostic tests in unfit patients who appear to have advanced and widely metastatic disease is justifiable and may reflect rapid clinical deterioration that prompts the treating physician to limit diagnostic procedures early. This may also explain why oncologists made no attempt to clarify the unspecified histology of ‘malignant neoplasm’ in untreated patients. However, we cannot rule out that treating doctors do not recommend completion of diagnostic investigations because of pessimism, lack of timely access to diagnostic investigations, or lack of knowledge regarding available treatment options. The likelihood of receiving anticancer pharmacotherapy increased with younger age, reduced comorbidity score, having a histology of squamous, neuroendocrine, or small-cell carcinomas, and other more specific histologies (compared with adenocarcinoma). Although there were no notable disparities among patients who received histologic confirmation based on race or urban/rural settings, histologic confirmation was slightly less common among those with a higher poverty index. A higher poverty index was also found to be negatively associated with receipt of anticancer pharmacotherapy. Further research is required to better understand referral pathways to receive more complete diagnostic work-up and treatment among patients with CUP.

Patients who did not receive a full diagnostic work-up had shorter median overall survival, particularly with increasing age. The high early and overall mortality among patients who did not receive histologic confirmation suggests that these patients in general were nevertheless suffering from cancer; although without histologic confirmation, some of these individuals may have had more easily treatable cancers than CUP, another treatable diagnosis mimicking cancer on imaging, or a relapse of a pre-existing malignancy that might have been missed [29,30,31]. Given that the time to treatment was similar to the median survival time in this population, it is not surprising that a shorter median survival was observed in untreated patients, although no causal relationship can be inferred based on this study. Among treated patients, over half received chemotherapy but often only few cycles were possible, suggesting a lack of response or poor tolerance to existing therapies, or limited availability of more specific options. Very few patients received immunotherapy or targeted treatments, likely due to a lack of comprehensive testing for predictive markers and molecular targets, respectively, as well as a lack of approved treatments or guideline-recommended treatments for CUP in the study period.

The strengths of this study include the use of population-based representative data for patients with CUP aged ≥ 66 years old and broad examination of claims-based diagnostic activities. Limitations include: (1) lack of younger patients (although these account for only a minority of patients with CUP overall and in the SEER database [22]); (2) some diagnostic procedures and treatments may have been missed if covered by non-Medicare (private) insurance; and (3) lack of detailed clinical data (e.g., number of metastatic organs involved, results of clinical examinations, and details of clinical interventions). This limited our ability to verify the receipt of state-of-the-art diagnostic work-up fully [28] and thoroughly investigate clinical factors known to affect survival [32,33,34]. We utilized information from Part D prescription medication files when analyzing treatment receipt. However, 32% of patients in the study did not have sufficient Part D coverage (i.e., <12 months prior to CUP diagnosis, or up until 31 December 31 2016 post-CUP diagnosis, or until death if that occurred earlier), and we potentially might have missed some treatments (e.g., oral chemotherapy agents). Nonetheless, sensitivity analysis restricted to the population of patients with Part D coverage did not find any meaningful differences in baseline diagnostic work-up or treatment characteristics. Some exclusion criteria-based selection bias may have occurred due to some patients having CUP identified by death certificate or autopsy only, and some being without sufficient Medicare part A & B enrollment time prior to diagnosis, although both groups were small (~10%).

## 5. Conclusions

This SEERM study suggests that further research is needed to understand why many patients given a diagnosis of CUP do not receive a complete diagnostic work-up or treatment. A substantial unmet need exists for timely access to more accurate and rapid diagnostic work-ups, as well as more effective treatments for patients diagnosed with CUP. Baseline diagnostic work-up was performed on patients who subsequently received anticancer pharmacotherapy, and significantly better survival outcomes were seen in patients who were well enough and able to access guideline-recommended diagnostic work-up. However, poor general health status of patients or aggressive, advanced disease may lead the treating physician to cease diagnostic procedures prior to establishing a diagnosis. Furthermore, among those who received treatment, ~45% received up to 4 days of treatment only, implying that lack of response or poor tolerance of existing therapies is common and that more specific treatment options are rarely available. High-resolution clinical studies on CUP are needed to provide further insights into the unmet needs observed in this study.

## Figures and Tables

**Table 1:** Baseline demographics ^a^ and clinical characteristics of patients with CUP, overall, and by receipt of anticancer pharmacotherapy.

	Overall		Histologically Confirmed		Not Histologically Confirmed ^b^	
**Characteristic**	*n*	%	*n*	%	*N*	%
Total number of patients	4562	100.0	2813	100.0	1749	100.0
**Age at CUP diagnosis, years**						
Median (IQR)	80	73.0–86.0	78	72.0–84.0	84	78.0–89.0
**Age groups, years**						
66–74	1334	29.2	1018	36.2	316	18.1
75–84	1728	37.9	1133	40.3	595	34.0
≥85	1500	32.9	662	23.5	838	47.9
**Sex**						
Female	2453	53.8	1449	51.5	1004	57.4
Male	2109	46.2	1364	48.5	745	42.6
**Race**						
White	3936	86.3	2414	85.8	1522	87.0
Black	358	7.9	220	7.8	138	7.9
Other/Unknown	268	5.8	179	6.4	89	5.1
**Year of CUP diagnosis**						
2013	1601	35.1	999	35.5	602	34.4
2014	1523	33.4	931	33.1	592	33.9
2015	1438	31.5	883	31.4	555	31.7
**Poverty indicator, ^c^ %**						
0–<5	1024	22.5	664	23.6	360	20.6
5–<10	1072	23.5	671	23.9	401	22.9
10–<20 ^d^	1341	29.4	807	28.7	534	30.5
20–100	1125	24.7	671	23.9	454	26.0
Unknown/Missing	S	S	S	S	S	S
**Urban vs. rural**						
All urban ^d^	2667	58.5	1673	59.5	994	56.8
Mostly urban	1063	23.3	633	22.5	430	24.6
Mostly rural	392	8.6	245	8.7	147	8.4
All rural	440	9.6	262	9.3	178	10.2
Unknown/Missing	S	S	S	S	S	S
**Patient status at study end**						
Died	4163	91.3	2479	88.1	1684	96.3
Lost to follow-up	399	8.8	334	11.9	65	3.7
	**Overall**		**Treated ^e^**		**Untreated ^f^**	
**Characteristic**	*n*	%	*n*	%	*N*	%
Total	4562	100.0	621	100.0	3941	100.0
**NCI comorbidity score ^g^**						
0	1446	31.7	302	48.6	1144	29.0
Low (1–<2)	1035	22.7	163	26.3	872	22.1
Middle (2–<4)	932	20.4	96	15.5	836	21.2
High (≥4)	1149	25.2	60	9.7	1089	27.6
**Basis of diagnostic confirmation ^h^**						
Histologic confirmation ^i^	2813	61.7	604	97.3	2209	56.1
Imaging only ^j^	1336	29.3	13	2.1	1323	33.6
Other ^k^	202	4.4	S	S	199	5.1
Clinical only ^l^	211	4.6	S	S	210	5.3
**Histology (among those with histologic confirmation)**						
Adenocarcinoma	1149	25.2	207	33.3	942	23.9
Carcinoma, NOS ^m^	479	10.5	77	12.4	402	10.2
Squamous-cell carcinoma, NOS	312	6.8	97	15.6	215	5.5
Neuroendocrine carcinoma	177	3.9	69	11.1	108	2.7
Small-cell carcinoma, NOS	94	2.1	S	S	S	S
Neoplasm, malignant	148	3.2	S	S	S	S
Other histology	454	10.0	117	18.8	337	8.6
**Not histologically confirmed**	1749	38.3	17	2.7	1732	44.0

CUP, cancer-of-unknown-primary-origin; IQR, interquartile range; NCI, National Cancer Institute; NOS, not otherwise specified; SEER, Surveillance, Epidemiology, and End Results; SEERM, Surveillance, Epidemiology, and End Results–Medicare. S denotes suppressed data based on NCI guidelines for confidentiality. ^a^ Based on SEERM cancer registry data. ^b^ Identification via imaging only (radiology/other imaging techniques without histologic confirmation), other (positive laboratory test/marker study; direct visualization without histologic confirmation), or clinical diagnosis only (other than imaging only or other). ^c^ The poverty indicator denotes the proportion of the population that lives below the federal poverty line in the patient’s census tract. A total of 0%–<5% poverty represents wealthier census tracts where most people are above the poverty line and 20% to 100% poverty groups represent less wealthy tracts. ^d^ Suppressed rows are added to the most populous row here for privacy reasons. ^e^ Patients receiving anticancer pharmacotherapy. ^f^ Patients not receiving anticancer pharmacotherapy. ^g^ Score based on the number of comorbid conditions. ^h^ Based on SEER registry data. ^I^ Positive histology (for a malignant tumor) only, positive cytology only, or positive histology plus positive immunophenotyping and/or positive genetic studies. ^j^ Radiology and other imaging techniques without histologic confirmation. ^k^ Positive laboratory test/marker study; direct visualization without histologic confirmation. ^l^ Clinical diagnosis only (other than imaging only or other). ^m^ Carcinoma NOS was documented where there was a lack of information available in the files of the patient.

**Table 2:** Characteristics of patients with CUP, overall, and by receipt of anticancer pharmacotherapy.

	Overall		Treated ^a^			
**Characteristic**	***n***	**%**	***n***	**%**		
Total	4562	100.0	621	100.0		
**Cancer therapies applied**						
Radiotherapy:						
No	4007	87.8	366	58.9		
Yes	555	12.2	255	41.1		
Surgery:						
No	4272	93.6	521	83.9		
Yes	290	6.4	100	16.1		
**Number of patients with ≥1 claim for types of pharmacotherapy ^b^**						
Chemotherapy only	369	8.1	369	59.4		
Immunotherapy ^c^	22	0.5	22	3.5		
Targeted therapy ^c^	99	2.2	99	15.9		
Antineoplastic infusion code only ^d^	58	1.3	58	9.3		
Other treatments ^e^	95	2.1	95	15.3		
**Treated Patients Only**						
**Median number of days from CUP diagnosis until pharmacotherapy treatment initiation**	***n***	**Median**	**Q1**	**Q3**	**Minimum**	**Maximum**
Overall	621	41	24	70	1	1337
**By age group, years**						
66–74	319	41	24	66	1	1337
75–84	244	44	24	85	1	1240
≥85	58	37	20	71	3	579
**Number of days of treatment ^f^**	***n***	**%**	***n***	**%**		
1 day	114	18.4	114	18.4		
2–4 days	169	27.2	169	27.2		
5–9 days	151	24.3	151	24.3		
10–19 days	103	16.6	103	16.6		
≥20 days	84	13.5	84	13.5		

CUP, cancer-of-unknown-primary-origin. ^a^ Patients receiving anticancer pharmacotherapy. ^b^ Any time in follow-up, alone, in parallel, or in sequential combination with other agents, unless otherwise indicated. ^c^ Nine patients received both immunotherapy and targeted therapy. ^d^ Claim for antineoplastic infusion only—drug names were not provided with the claim. When drug names were provided, these claims were not accounted for separately. ^e^ Treatments other than chemotherapy, targeted therapy, and immunotherapy, such as hormonal or supportive care therapies (e.g., denosumab). ^f^ Unique days with treatment administered in the follow-up period, not duration of treatment (only days where patients had received anticancer pharmacotherapy treatment were included).

**Table 3:** Multivariate logistic regression assessing the odds of receiving pharmacotherapy among patients with histologic confirmation only.

Variable	Categories (Event = Receiving Pharmacotherapy)	Odds Ratio	Lower 95% CI	Upper 95% CI	*p* Value
Age group, years	75–84 vs. **66–74**	**0.61**	0.50	0.76	**<0.0001**
	≥85 vs. **66–74**	**0.23**	0.16	0.31	**<0.0001**
Sex	Male vs. **Female**	1.10	0.90	1.34	0.360
Race	Black vs. **White**	0.78	0.51	1.18	0.236
	Other vs. **White**	1.16	0.76	1.77	0.495
	Unknown vs. **White**	0.80	0.28	2.33	0.685
Year of treatment	2013 vs. **2015**	1.19	0.94	1.51	0.152
	2014 vs. **2015**	1.01	0.79	1.29	0.960
Comorbidity score	Low: 1–<2 vs. **0**	**0.66**	0.52	0.84	**0.0001**
	Mid: 2–<4 vs. **0**	**0.47**	0.36	0.62	**<0.0001**
	High: ≥4 vs. **0**	**0.25**	0.18	0.35	**<0.0001**
Extended work-up ^a^	At least one vs. **all three**	**0.51**	0.38	0.69	**<0.0001**
	None vs. **all three**	**0.05**	0.01	0.22	**<0.0001**
Histology	Carcinoma, NOS vs. **adenocarcinoma**	0.83	0.62	1.13	0.237
	Squamous-cell carcinoma, NOS vs. **adenocarcinoma**	**2.11**	1.54	2.87	**<0.0001**
	Neuroendocrine carcinoma vs. **adenocarcinoma**	**2.54**	1.77	3.64	**<0.0001**
	Small-cell carcinoma, NOS vs. **adenocarcinoma**	**2.21**	1.35	3.63	**0.002**
	Neoplasm, malignant vs. **adenocarcinoma**	**0.42**	0.21	0.87	**0.019**
	Other histology vs. **adenocarcinoma**	**1.42**	1.08	1.87	**0.012**
Urbanicity	Mostly urban vs. **all urban**	0.97	0.76	1.24	0.804
	All rural vs. **all urban**	0.82	0.57	1.18	0.283
	Mostly rural vs. **all urban**	0.95	0.67	1.35	0.783
Poverty indicator, %	5–<10 vs. **0–<5**	0.95	0.73	1.25	0.716
	10–<20 vs. **0–<5**	**0.65**	0.50	0.86	**0.002**
	20–100 vs. **0–<5**	**0.67**	0.50	0.90	**0.008**
	Unknown vs. **0–<5**	0.83	0.07	10.6	0.885

CI, confidence interval; CUP, cancer-of-unknown-primary-origin; NOS, not otherwise specified. ^a^ In treated patients, work-up was considered until a maximum of 30 days after CUP diagnosis or until treatment start (anticancer pharmacotherapy) if that happened earlier.

## Data Availability

In accordance with the policy of NCI, the authors are not able to provide SEER–Medicare data to any other individual or investigator. Investigators interested in the data should contact NCI to discuss arrangements for ordering an extra copy of the dataset.

## Supplementary Materials

The following supporting information can be downloaded at: https://www.mdpi.com/article/10.3390/cancers14122905/s1, Information on how complex code lists were compiled for this study; Figure S1: Claim volumes for selected diagnostic work-ups among patients diagnosed with CUP, before and after the CUP diagnosis, in 2-week periods.^a^ Selected diagnostic procedures include (A) biopsy, (B) immunohistochemistry, and (C) imaging; Table S1: Attrition table showing the creation of the analysis cohorts; Table S2: Attrition table showing upfront administrative exclusions; Table S3: Baseline work-up among patients with CUP, by age and treatment, between 365 days before and 30 days post-CUP diagnosis (index); Table S4: Survival estimates for patients with CUP (from CUP diagnosis date, overall, and by treatment and age); Table S5: Overall survival by number of days with treatment. [35,36,37,38,39] have been cited in the supplementary materials.
