# Supplementary material for: Cancer-of-Unknown-Primary-Origin: A SEER–Medicare Study of Patterns of Care and Outcomes among Elderly Patients in Clinical Practice
Source: Cancers (Basel). 2022 Jun 13;14(12):2905. doi: 10.3390/cancers14122905 (PMC9221531; doi:10.3390/cancers14122905)
Supplement: Supplementary file 1 [file cancers-14-02905-s001.zip › cancers-1715537-supplementary.pdf]

SUPPLEMENTARY MATERIAL

INFORMATION ON HOW COMPLEX CODE LISTS WERE COMPILED FOR THIS STUDY

Code lists for anticancer pharmacotherapies

We note that National Drug Code (NDC) numbers and Healthcare Common Procedure Coding System (HCPCS) codes, which are used to identify treatments in administrative claims databases, are updated regularly; we recommend that the code lists be created based on up-to-date look-up files at the time of initiating a new research study.

First, we compiled a list of antineoplastic agents from publicly available sources [1]. Their corresponding drug classes were assigned based on treatment look-up tables of internally licensed, commercially available claims data sources. The complete list of generic (or non-proprietary) names and their classifications in our study are shared below and may be re-used.

In a second step, we generated the corresponding NDC and HCPCS codes based on string searches of product generic names in NDC look-up tables and string searches of procedure descriptions for HCPCS codes. The NDC and HCPCS coding systems are publicly available [2,3]. Users can identify NDC numbers of interest based on searching for the generic names provided in our table on pages 3–14. With regard to the HCPCS codes, users can select the appropriate codes based on the code description contained in the look-up file, for example: *J9060 - Injection, cisplatin, powder or solution, 10 mg* corresponds to cisplatin.

Code lists for diagnostics (biopsy, imaging, immunohistochemistry)

Similarly, the procedure code lists used to identify claims for disease work-up (biopsy, imaging, and immunohistochemistry) were generated by conducting text searches of the following terms using an internally licensed Common Procedural Terminology (CPT) look-up file, which is maintained by the American Medical Association, and subsequently removing terms not indicative of the procedures of interest after additional review:

- Biopsy: "biopsy", "diagnostic", "excision", "extraction";
- Imaging: "magnetic resonance", "positron emission tomography", "computed tomography";
- Immunohistochemistry: "immunohistochemistry"

Additionally, codes were validated by using payer coding guidance [4,5] and a medical coder. Finally, the most frequently occurring codes for each type of procedure were assessed in the data as part of an exploratory analysis during the study to ensure that all relevant codes were included.

Alphabetically ordered list of medications and their drug classes used in the study

Generic Name	Drug Class
abemaciclib	targeted therapy
abiraterone	hormone therapy
acalabrutinib	targeted therapy
ado-trastuzumab emtansine	targeted therapy
afatinib	targeted therapy
aldesleukin	immunotherapy
alectinib	targeted therapy
alemtuzumab	targeted therapy
alpelisib	targeted therapy
altretamine	chemotherapy
anastrozole	hormone therapy
apalutamide	hormone therapy
arsenic	chemotherapy
asparaginase	chemotherapy
atezolizumab	immunotherapy
avelumab	immunotherapy

axicabtagene ciloleucel	immunotherapy
axitinib	targeted therapy
azacitidine	chemotherapy
BCG vaccine	immunotherapy
belinostat	targeted therapy
bendamustine	chemotherapy
bevacizumab	targeted therapy
bevacizumab-awwb	targeted therapy
bexarotene	chemotherapy
bicalutamide	hormone therapy
binimetinib	targeted therapy
bleomycin	chemotherapy
blinatumomab	targeted therapy
bortezomib	targeted therapy
bosutinib	targeted therapy
brentuximab vedotin	targeted therapy
brigatinib	targeted therapy
busulfan	chemotherapy

cabazitaxel	chemotherapy
cabozantinib	targeted therapy
capecitabine	chemotherapy
carboplatin	chemotherapy
carfilzomib	targeted therapy
carmustine	chemotherapy
cemiplimab	immunotherapy
ceritinib	targeted therapy
cetuximab	targeted therapy
chlorambucil	chemotherapy
cisplatin	chemotherapy
cladribine	chemotherapy
clofarabine	chemotherapy
cobimetinib	targeted therapy
cobimetinib	targeted therapy
copanlisib	targeted therapy
crizotinib	targeted therapy
cyclophosphamide	chemotherapy

cytarabine	chemotherapy
cytarabine liposomal	chemotherapy
dabrafenib	targeted therapy
dacarbazine	chemotherapy
dacomitinib	targeted therapy
dactinomycin	chemotherapy
daratumumab	targeted therapy
dasatinib	targeted therapy
daunorubicin	chemotherapy
daunorubicin/cytarabine liposomal	chemotherapy
decitabine	chemotherapy
degarelix	hormone therapy
diethylstilbestrol	hormone therapy
docetaxel	chemotherapy
doxorubicin	chemotherapy
doxorubicin pegylated liposomal	chemotherapy
durvalumab	immunotherapy
duvelisib	targeted therapy

elotuzumab	targeted therapy
enasidenib mesylate	targeted therapy
encorafenib	targeted therapy
entrectinib	targeted therapy
enzalutamide	hormone therapy
epirubicin	chemotherapy
erdafitinib	targeted therapy
eribulin	chemotherapy
erlotinib	targeted therapy
estramustine	hormone therapy
etoposide	chemotherapy
everolimus	targeted therapy
exemestane	hormone therapy
fedratinib	targeted therapy
floxuridine	chemotherapy
fludarabine	chemotherapy
fluorouracil	chemotherapy
flutamide	hormone therapy

fulvestrant	hormone therapy
gefitinib	targeted therapy
gemcitabine	chemotherapy
gemtuzumab ozogamicin	targeted therapy
gilteritinib	targeted therapy
goserelin	hormone therapy
histrelin	hormone therapy
hydroxyurea	chemotherapy
ibrutinib	targeted therapy
idarubicin	chemotherapy
idelalisib	targeted therapy
ifosfamide	chemotherapy
imatinib	targeted therapy
inotuzumab ozogamicin	targeted therapy
interferon alfa-2a	immunotherapy
interferon alfa-2b	immunotherapy
ipilimumab	immunotherapy
irinotecan	chemotherapy

irinotecan liposomal	chemotherapy
ivosidenib	targeted therapy
ixabepilone	chemotherapy
ixazomib	targeted therapy
lapatinib	targeted therapy
larotrectinib	targeted therapy
lenalidomide	targeted therapy
lenvatinib	targeted therapy
letrozole	hormone therapy
leuprolide	hormone therapy
lomustine	chemotherapy
lorlatinib	targeted therapy
mechlorethamine	chemotherapy
medroxyprogesterone	hormone therapy
megestrol	hormone therapy
melphalan	chemotherapy
mercaptopurine	chemotherapy
methotrexate	chemotherapy

midostaurin	targeted therapy
mitomycin	chemotherapy
mitotane	hormone therapy
mitoxantrone	chemotherapy
mogamulizumab	targeted therapy
necitumumab	targeted therapy
neratinib	targeted therapy
nilotinib	targeted therapy
nilutamide	hormone therapy
nintedanib	targeted therapy
niraparib	targeted therapy
nivolumab	immunotherapy
obinutuzumab	targeted therapy
ofatumumab	targeted therapy
olaparib	targeted therapy
olaratumab	targeted therapy
osimertinib	targeted therapy
oxaliplatin	chemotherapy

paclitaxel	chemotherapy
paclitaxel protein-bound	chemotherapy
palbociclib	targeted therapy
panitumumab	targeted therapy
panobinostat	targeted therapy
pazopanib	targeted therapy
pegaspargase	chemotherapy
peginterferon alfa-2b	immunotherapy
pembrolizumab	immunotherapy
pemetrexed	chemotherapy
pentostatin	chemotherapy
pertuzumab	targeted therapy
polatuzumab	targeted therapy
pomalidomide	targeted therapy
ponatinib	targeted therapy
pralatrexate	chemotherapy
procarbazine	chemotherapy
ramucirumab	targeted therapy

regorafenib	targeted therapy
ribociclib	targeted therapy
ribociclib/letrozole	targeted therapy
rituximab	targeted therapy
rituximab/hyaluronidase	targeted therapy
romidepsin	targeted therapy
rucaparib	targeted therapy
ruxolitinib	targeted therapy
siltuximab	targeted therapy
sipuleucel-t	immunotherapy
sonidegib	targeted therapy
sorafenib	targeted therapy
sunitinib	targeted therapy
talazoparib	targeted therapy
talimogene laherparepvec	immunotherapy
tamoxifen	hormone therapy
temozolomide	chemotherapy
temsirolimus	targeted therapy

thalidomide	targeted therapy
thioguanine	chemotherapy
thiotepa	chemotherapy
tisagenlecleucel	immunotherapy
topotecan	chemotherapy
toremifene	hormone therapy
trabectedin	chemotherapy
trametinib	targeted therapy
trastuzumab	targeted therapy
trastuzumab/hyaluronidase-oysk	targeted therapy
trastuzumab-anns	targeted therapy
tretinoin	chemotherapy
trifluridine/tipiracil	chemotherapy
triptorelin	hormone therapy
valrubicin	chemotherapy
vandetanib	targeted therapy
vemurafenib	targeted therapy
venetoclax	targeted therapy

vinblastine	chemotherapy
vincristine	chemotherapy
vincristine liposomal	chemotherapy
vinorelbine	chemotherapy
vismodegib	targeted therapy
vorinostat	targeted therapy
ziv-aflibercept	targeted therapy

Figure S1. Claim volumes for selected diagnostic work-ups among patients diagnosed with CUP, before and after the CUP diagnosis, in 2-week periods.[a] Selected diagnostic procedures include (**A**) biopsy, (**B**) immunohistochemistry, and (**C**) imaging.

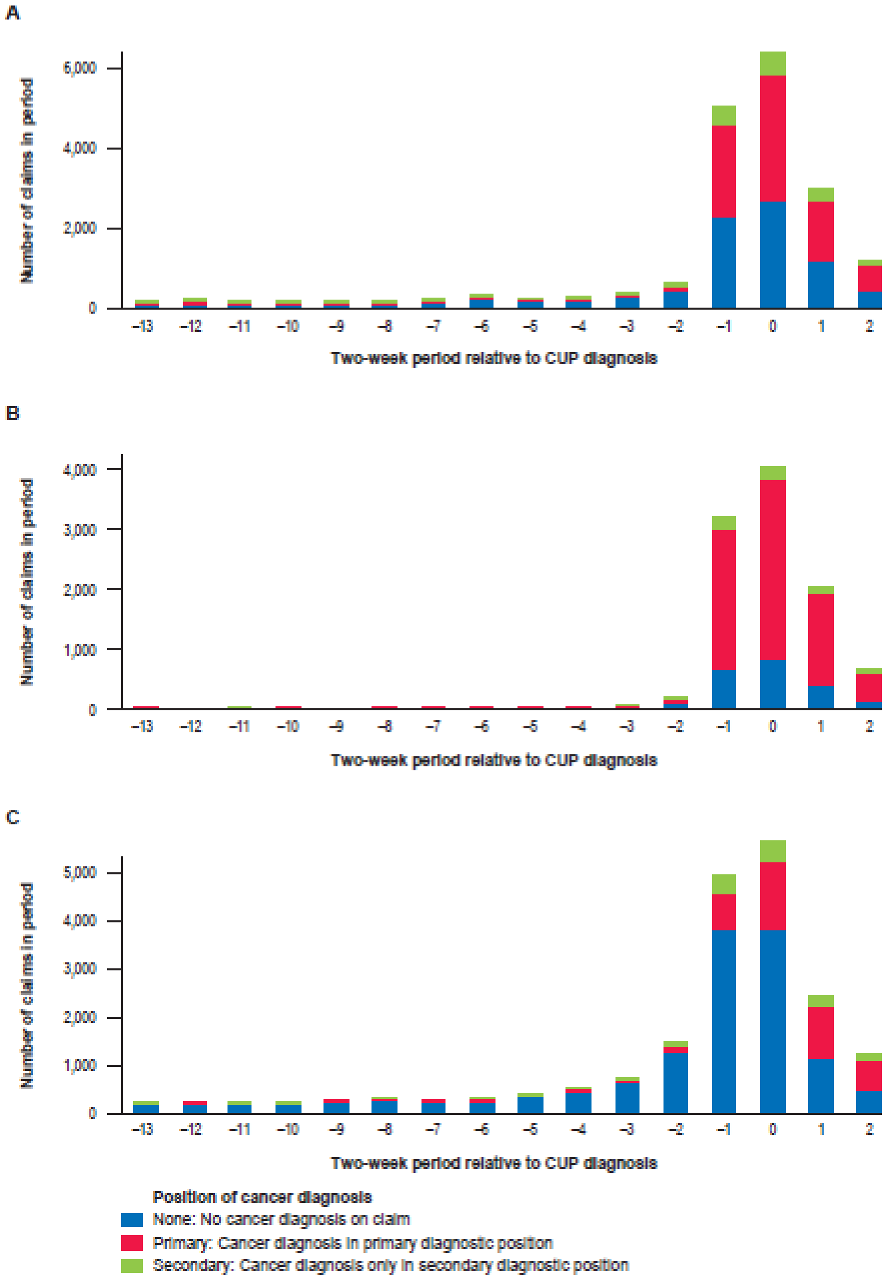

CUP, cancer-of-unknown-primary-origin.

[a] Data for the 26 weeks before CUP diagnosis is shown.

Table S1. Attrition table showing the creation of the analysis cohorts.

Step	Attrition Reason	Patients	
		n	%
	Study inclusion criteria		
	Patients with a first malignant CUP diagnosis in SEERM cancer registry files at ≥66 years old (1/1/13–31/12/15)	6,116	100.0
	**Study exclusion criteria**		
1	CUP identified exclusively by death certificate or autopsy	657	10.7
2	Patients were diagnosed with a subsequent (separate primary) cancer following their diagnosis of CUP	92	1.5
3	Patients with insufficient Medicare Part A & B enrollment (inpatient, doctor's services, and outpatient cover)[a]	578	9.5
4	Patients with a cancer treatment claim in Medicare prior to CUP diagnosis[b]	227	3.7
C1	Patients in the study cohort for analysis	4,562	74.6

CUP, cancer-of-unknown-primary-origin; SEERM, Surveillance, Epidemiology, and End Results – Medicare.

[a] <12 months prior to CUP diagnosis, or up until December 31, 2016 post-CUP diagnosis, or until death if that occurred earlier.

[b] In the 12 months prior to the patient's CUP diagnosis date.

Table S2. Attrition table showing upfront administrative exclusions.

Step	Attrition Reason	Patients	
		n	%
	**Starting number of patients**<br>(all patients included in the obtained SEERM data extract)	169,595	100.0
	**Upfront administrative exclusions**	**Exclusions**	
0.1	Patients without agreement on date of birth between SEER and Medicare (DOB_FLG)	2,342	1.4
0.2	Patients without agreement on date of death between SEER and Medicare (DOD_FLG)	728	0.4
0.3	Individuals enrolled in an HMO[a] (Medicare C) in the 12-month period prior to cancer diagnosis or following diagnosis	26,240	15.5

DOB_FLG, date of birth flag; DOD_FLG, date of death flag; HMO, health maintenance organization; SEER, Surveillance, Epidemiology, and End Results; SEERM, Surveillance, Epidemiology, and End Results – Medicare.

[a] HMOs are closed healthcare provision systems who only provide care by and through contracted physicians and providers. Claims for patients cared for in these organizations are not submitted and are therefore not visible to Medicare.

Table S3. Baseline work-up among patients with CUP, by age and treatment, between 365 days before and 30 days post-CUP diagnosis (index).

	**Treated Patients *n* = 621**						**Untreated Patients *n* = 3,941**					
	Age 66–74		Age 75–84		Age ≥85		Age 66–74		Age 75–84		Age ≥85	
**Work-Up Type**	*n*	%	*n*	%	*n*	%	*n*	%	*n*	%	*n*	%
Total	319	100	244	100	58	100	1,015	100	1,484	100	1,442	100
Biopsy + Imaging + IHC	272	85.3	199	81.6	45	77.6	603	59.4	788	53.1	474	32.9
Biopsy + Imaging	31	9.7	30	12.3	S	S	128	12.6	234	15.8	281	19.5
Biopsy + IHC	S	S	S	S	S	S	18	1.8	16	1.1	26	1.8
IHC + Imaging	S	S	0	0.0	S	S	S	S	S	S	S	S
IHC alone	S	S	0	0.0	0	0.0	0	0.0	0	0.0	0	0.0
Imaging alone	S	S	11	4.5	S	S	193	19.0	380	25.6	560	38.8
Biopsy alone	S	S	0	0.0	0	0.0	S	S	S	S	16	1.1
None	S	S	S	S	0	0.0	63	6.2	60	4.0	81	5.6

CUP, cancer-of-unknown-primary-origin; IHC, immunohistochemistry; NCI, National Cancer Institute.
S denotes suppressed data based on NCI guidelines.

Table S4.

Survival estimates for patients with CUP (from CUP diagnosis date, overall, and by treatment and age).

	Median Survival						Milestone Survival (%)					
	Cohort	*n*	Median (months)	Lower 95% CI	Upper 95% CI	*p* value[a]	Month 1	Month 2	Month 6	Month 12	Month 24	Month 36
	Overall	4,562	1.2	1.2	1.3		55.8	29.4	20.3	13.7	9.2	7.1
**Treatment (pharmacotherapy)**	Yes	621	9.5	8.2	10.5	<0.0001	96.6	78.4	62.5	42.5	26.6	19.7
	No	3,941	1.0	0.9	1.0		49.4	21.7	13.7	9.1	6.5	5.2
**Age group**	66–74	1,334	1.6	1.4	1.7	<0.0001[b]	63.3	36.7	27.0	19.2	14.8	12.4
**Treatment (pharmacotherapy)**	Yes	319	9.5	7.9	10.9	<0.0001	95.9	78.4	62.1	41.1	29.4	25.5
	No	1,015	1.1	1.0	1.2		53.0	23.7	16.0	12.3	10.2	8.3
**Age group**	75–84	1,728	1.2	1.1	1.3		55.4	28.4	20.1	13.7	9.1	6.9
**Treatment (pharmacotherapy)**	Yes	244	9.2	7.7	12.0	<0.0001	97.5	78.7	62.7	43.9	24.1	15.6
	No	1,484	1.0	0.9	1.0		48.5	20.1	13.1	8.8	6.8	5.7
**Age group**	≥85	1,500	1.0	0.9	1.1		49.7	24.1	14.7	8.7	4.4	2.8
**Treatment (pharmacotherapy)**	Yes	58	10.5	6.2	13.7	<0.0001	96.6	77.6	63.8	44.8	22.9	8.9
	No	1,442	0.9	0.9	1.0		47.9	21.9	12.7	7.2	3.6	2.6

CI, confidence interval; CUP, cancer-of-unknown-primary-origin.

[a] Log-rank test.

[b] Comparing across age groups, independent of treatment.

Table S5.

Overall survival by number of days with treatment.

Survival by Timeframe of Treatment[a], Days	*n*	Events	Median (Months)	Lower 95% CI	Upper 95% CI
0	3,941	3,696	1.0	0.9	1.0
1	114	107	2.1	1.8	2.6
2–4	169	142	4.7	3.6	5.5
5–9	151	105	11.4	9.0	14.5
10–19	103	62	17.4	13.3	20.6
≥20	84	51	20.8	20.6	25.7

CI, confidence interval.

[a] Unique days with treatment administered in the follow-up period; not duration of treatment.

References

1. National Cancer Institute. A to Z List of Cancer Drugs. Available online: https://www.cancer.gov/about-cancer/treatment/drugs (accessed on 20 May 2022).
2. U.S. Food and Drug Administration (FDA). National Drug Code Directory. Available online: https://www.accessdata.fda.gov/scripts/cder/ndc/index.cfm (accessed on 20 May 2022).
3. Healthcare Common Procedure Coding System. Coding System. Available online: https://www.cms.gov/files/zip/july-2022-alpha-numeric-hcpcs-file.zip (accessed on 20 May 2022).
4. Aetna. Positron Emission Tomography (PET). Available online: http://www.aetna.com/cpb/medical/data/1_99/0071.html (accessed on 20 May 2022).
5. American Medical Association. National Correct Coding Initative Policy Manual for Medicare Services. Available online: https://www.cms.gov/files/document/chapter10cptcodes80000-89999final11.pdf (accessed on 20 May 2022).
